# A Quiz Compend of Gynecology

**Published:** 1899-11

**Authors:** 


A QUIZ COMPEND OF GYNECOLOGY. By W. H. Wells, M.D., Adjunct Professor of Obstetrics and Diseases of Infancy in the Philadelphia Polyclinic. P. Blakiston's Sons & Co., Philadelphia. Price 80c.

This is a second edition of the Compend on Gynecology, and like all of the series is thoroughly up to date. The Messrs. Blakiston have given to the medical student a most excellent series of Compends. This little book is well illustrated, and, for its size, is exceedingly thorough. As a Compend it is exceedingly meritorious. R.

